# Inhibition of Glutamine Synthetase: A Potential Drug Target in *Mycobacterium tuberculosis*

**DOI:** 10.3390/molecules190913161

**Published:** 2014-08-26

**Authors:** Sherry L. Mowbray, Muthu K. Kathiravan, Abhishek A. Pandey, Luke R. Odell

**Affiliations:** 1 Department of Cell and Molecular Biology, Science for Life Laboratory, Uppsala University, Biomedical Center, Box 596, SE-751 24 Uppsala, Sweden; 2 Seven Hills College of Pharmacy, Venkatramapuram (Via) Tanapalli, 517561 Tirupati, India; 3 Department of Pharmaceutical Chemistry, Sinhgad College of Pharmacy, Vadgaon (BK), 411041 Pune, India; 4 Organic Pharmaceutical Chemistry, Department of Medicinal Chemistry, Uppsala University, Biomedical Center, Box 574, SE-751 23 Uppsala, Sweden

**Keywords:** tuberculosis, glutamine synthetase, drug discovery, structure-activity relationship, *Mycobacterium tuberculosis*

## Abstract

Tuberculosis is an infectious disease caused by *Mycobacterium tuberculosis*. Globally, tuberculosis is second only to AIDS in mortality and the disease is responsible for over 1.3 million deaths each year. The impractically long treatment schedules (generally 6–9 months) and unpleasant side effects of the current drugs often lead to poor patient compliance, which in turn has resulted in the emergence of multi-, extensively- and totally-drug resistant strains. The development of new classes of anti-tuberculosis drugs and new drug targets is of global importance, since attacking the bacterium using multiple strategies provides the best means to prevent resistance. This review presents an overview of the various strategies and compounds utilized to inhibit glutamine synthetase, a promising target for the development of drugs for TB therapy.

## 1. Introduction

Tuberculosis (TB) is an infectious disease caused by *Mycobacterium tuberculosis*. The World Health Organization estimates that one-third of the world’s population is currently infected with *M. tuberculosis*, and that 1.3 million deaths result from these infections each year [1]. Globally, TB is second only to AIDS in infectious disease mortality, and indeed co-infection with the bacterium underlies a large fraction of the deaths attributed to HIV. TB is a disease with close socioeconomic links, since it attacks a disproportionate number of young adults, and flourishes in the context of poverty. It is curable in most cases, but the impractically long treatment schedules (generally 6–9 months) and unpleasant side effects of the current drugs often lead to poor patient compliance, which in turn has resulted in the emergence of multidrug resistant (MDR-TB) strains. More recently, the situation has been exacerbated by the increasing frequency of extensively drug-resistant (XDR) TB [2]. XDR-TB is characterized by resistance to at least two of the four first-line drugs (rifampicin and isoniazid), and to the fluoroquinolone and injectables (kanamycin, amikacin or capreomycin) that are second-line drugs. Furthermore, bacterial strains that are resistant to all existing TB drugs, causing totally drug-resistant (TDR) TB, have now been identified in Iran, India, South Africa and Italy [3]. Although only a limited number of TDR-TB cases have been confirmed to date, testing against a wide spectrum of drugs is not routinely performed, and so the reported numbers must be viewed as an underestimate of the true situation. The appearance of an incurable from of TB is a frightening prospect that has potentially disastrous consequences for humanity. It is particularly worrying because only one new TB drug (bedaquiline) has been approved by the FDA since the 1960s. Clearly, the development of new classes of anti-TB drugs is of global importance. New targets are a priority, since attacking the bacterium using multiple strategies provides the best means to prevent resistance [4].

Our main focus in this review is the glutamine synthetase (GS; EC 6.3.1.2) of *M. tuberculosis*, which catalyses the ATP-dependent condensation of ammonium and l-glutamate, thus forming l-glutamine, ADP, phosphate and a proton. *M. tuberculosis* in fact possesses four GS homologues, of which only one, the product of the *glnA1* gene (hereafter referred to as *Mt*GS), is highly expressed and essential for the growth of the bacteria both *in vitro* and *in vivo* [5]. In addition to its well-characterized role in bacterial nitrogen metabolism, *Mt*GS plays an important role in cell wall biosynthesis, specifically via the production of a poly-l-glutamate-glutamine component found exclusively in pathogenic mycobacteria [6,7]. Extracellular *Mt*GS may also affect pH modulation in phagosomes and consequently prevent phagosome-lysosome fusion [8]. Numerous studies indicate that inhibition of *Mt*GS is a feasible therapeutic strategy [7,9]. The extracellular location of the bulk of the enzyme [5] furthermore obviates problems associated with the uptake of compounds across the notoriously impermeable mycobacterial cell wall. A distantly related GS is found in eukaryotes. The role of the enzyme in mammals depends on tissue localization; in the brain, it regulates the levels of toxic ammonia and converts neurotoxic glutamate to glutamine, whereas in the liver, it is one of the enzymes responsible for the removal of ammonia. Therefore, useful inhibitors of the mycobacterial GS must not have significant effects on the mammalian enzyme [7].

**Figure 1:**
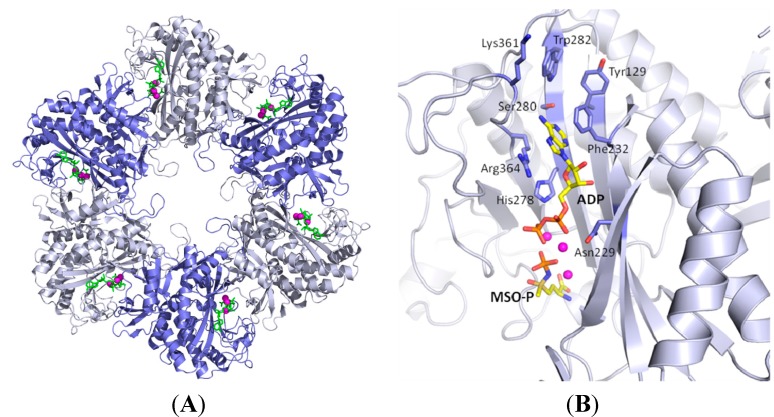
(**A**) X-ray structure of *Mt*GS (Protein Data Bank (PDB) entry 2BVC [13]) showing one hexameric ring. Ligands bound in the amino acid- and nucleotide-binding sites are shown in green, and the three ions of each active site as magenta spheres. (**B**) The active site of the A molecule bound to ADP and the inhibitor methionine sulfoximine phosphate (yellow carbons). The residues interacting with ADP are shown with blue carbons. The smaller contributions of the neighboring F molecule are not shown for clarity. Figures were prepared using MacPYMOL Version 1.3 [14].

Like other bacterial glutamine synthetases, *Mt*GS is a dodecamer formed of two hexameric rings stacked on top of each other; each active site includes contributions from two adjacent subunits within a ring [10]. The X-ray structure of a ligand-bound hexameric ring and a single active site are depicted in Figure 1. The activity of the enzyme is down-regulated under conditions of excess nitrogen through covalent addition of an AMP moiety to Tyr406 (*Mt*GS numbering) by the regulatory enzyme, GlnE. Since the subunits are essentially independent, the oligomer’s activity becomes progressively lower as the degree of adenylylation increases. Deadenylylation of *Mt*GS, also carried out by GlnE, releases this control under conditions of nitrogen limitation [11,12].

**Scheme 1:**
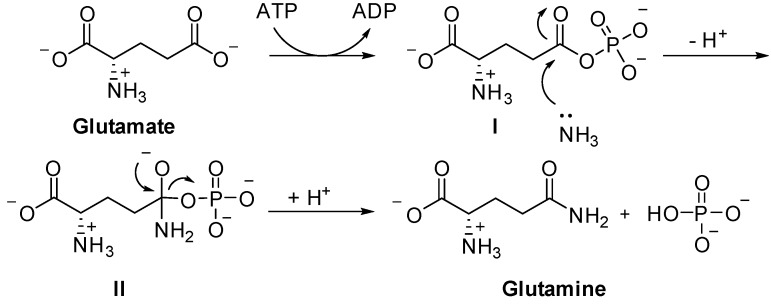
Glutamine synthetase catalyzes the formation of l-glutamine from l-glutamate.

To be in its active state, GS requires magnesium or manganese ions, located in three metal sites designated as n1-n3 [13]. In the first step of catalysis (Scheme 1), a tightly bound, activated intermediate, γ-glutamyl phosphate (I), is formed when the terminal phosphate of ATP is transferred to the carboxylate side chain of the substrate glutamate. In the second step, an enzyme-bound ammonium ion is deprotonated, forming ammonia that attacks the carbonyl carbon of γ-glutamyl phosphate to form a tetrahedral intermediate (II). The enzyme subsequently releases free phosphate and glutamine [15,16,17,18].

Comparisons of the available structures of *Mt*GS as well as those of *Salmonella typhimurium* GS (50% amino acid sequence identity) revealed that the bacterial enzymes have two major conformational states, described as relaxed (inactive) and taut (active) [13,15]. Most strikingly, a sliding motion of the β-strand starting at residue 214 (*Mt*GS numbering) of the β-barrel in the active site is correlated with metal binding. The strand shift creates a very different environment in the nucleotide, ammonium, and metal-binding sites, and so explains the large effects on enzyme activity.

## 2. Inhibitors of GS

The currently described inhibitors of GS can be divided into two broad categories. The first group are the small and highly polar amino acid analogues exemplified by two of the most widely used GS inhibitors, methionine sulfoximine (MSO), and phosphinothricin (PPT) [19]. These inhibitors target the amino acid-binding site, which is highly conserved in both bacterial and eukaryotic GSs. Consequently, selectivity issues may arise with this type of compound [13]. Inhibitors in the second class are typically larger, more hydrophobic heterocycles that compete with ATP. Importantly, the nucleotide-binding site is less conserved, and so inhibition via binding at this site is more likely to result in selective inhibitors [20].

### 2.1. Amino Acid Analogues

MSO (**1**), the first GS inhibitor described, was originally isolated from the maize protein zein after treatment with nitrogen trichloride [21]. It was later found that while MSO induces epilepsy in certain animals, primates are relatively insensitive [22]. MSO has been evaluated as an inhibitor of enzymes from a wide range of species including mammalian (e.g., sheep brain, *K*_i_ = 210 µM [23] and human, *K*_i_ = 1.19 mM [24]), plant (e.g., pea leaf, *K*_i_ = 161 µM [25]) and bacterial (e.g., *E. coli*, *K*_i_ = 2 µM [26]) GSs. Importantly, treatment of *M. tuberculosis* with MSO has been shown to inhibit both cell wall formation and bacterial growth (MIC = 8–12 µg/mL [27]), and the compound displayed *in vivo* antitubercular activity in a guinea pig model [7,9]. The *in vitro* potency of MSO against *Mt*GS was later found to be 51 ± 6 µM [28]. Unfortunately, a high rate of spontaneous resistance has been observed in bacteria treated with the compound (resulting from up-regulation of either GlnA1 or GlnA3 [29]), limiting its potential use as an anti-TB agent.

MSO initially binds as a competitive inhibitor and undergoes rapid phosphorylation by GS [24] producing the active form, methionine sulfoximine phosphate (**2**, MSO-P). MSO-P binds essentially irreversibly to the active site, preventing entry of the glutamate substrate [30]. The configuration of the two stereocenters has been shown to be important for inhibitory activity. The (*S*,*S*) diastereomer **3** is 10 times more potent than the (*S*,*R*) isomer **4** [1]; a recent X-ray analysis has uncovered the structural reasons for the enantiomer preference [13].

**Figure 2:**
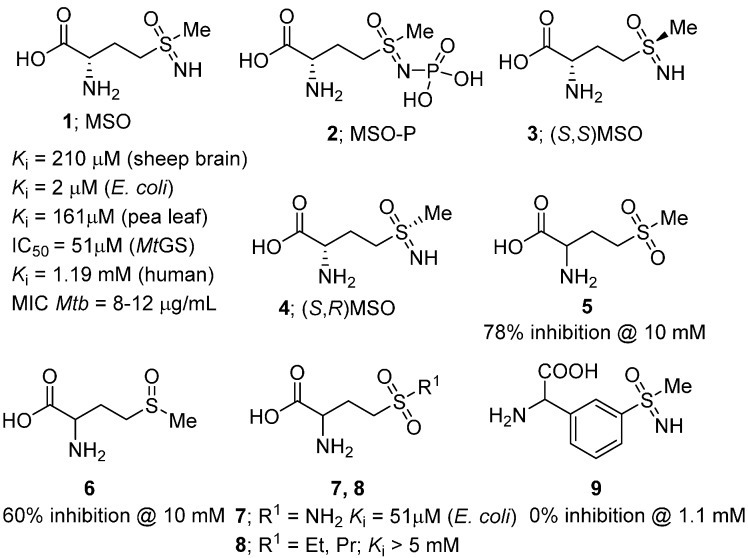
Structure and biological activities of MSO and analogues.

A number of MSO analogues have been designed and evaluated against GS (Figure 2). In contrast to MSO, the sulfone and sulfoxide analogues **5** and **6** were found to be weak, reversible GS inhibitors [30,31]. While the primary sulfonamide analogue **7** retained good levels of potency (*K*_i_ = 51 µM [18]), it was still significantly less active than MSO. A number of alkyl derivatives (**8**) such as methyl, ethyl and propyl, as well as phosphonate analogues of methionine sulfoximine have also been described, however, none were found to display greater inhibitory activity than MSO [32,33]. Finally, an aryl MSO analogue (**9**) has been synthesized where the amino acid side chain was replaced with a rigid phenyl ring [34]. The compound was prepared via a palladium-catalysed α-arylation of a protected glycine analogue, but unfortunately, did not inhibit *Mt*GS, even at a concentration of 1.1 mM.

**Figure 3:**
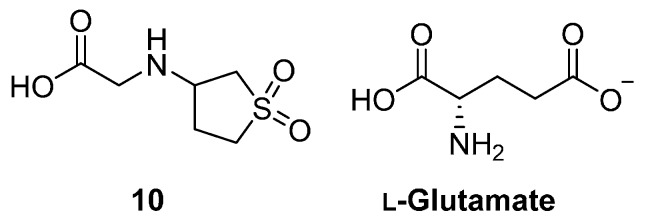
Cyclic sulfone **10** and the GS substrate, l-glutamate.

Recently, the cyclic sulfone **10** (Figure 3) was reported as an inhibitor of *M. tuberculosis* growth(MIC = 8–16 µg/mL [27]). This compound was identified via a 3D pharmacophore search based on its similarity to the essential molecule, L-glutamate. The pharmacophore contained three interaction points; the α-amino and carboxyl groups were represented by positive and negative ionization features, respectively, and a hydrogen-bond acceptor feature was included to account for the side-chain carboxylate. Due to the structural similarity between **10**, **1** and glutamate, the authors believed that **10** targets an enzyme involved in glutamine biosynthesis, presumably *Mt*GS, however this has not yet been confirmed.

**Figure 4:**
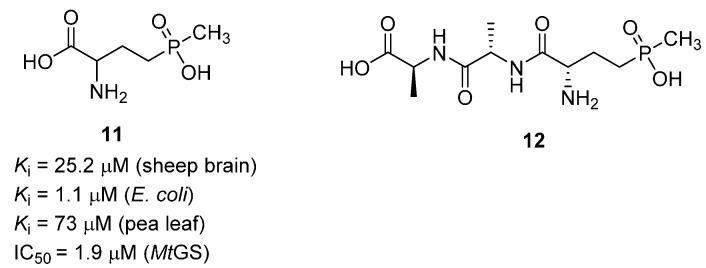
Structure and biological activity of phosphinothricin (**11**), and structure of its tripeptide precursor (**12**). References: sheep brain [37], *E. coli* [26], pea leaf [25] and *Mt*GS [28].

Phosphinothricin (**11**, PPT) is a potent GS inhibitor discovered independently by research groups in Germany and Japan in the early 1970s [35,36]. The compound was derived from *Streptomyces*, upon hydrolysis of the unusual tripeptide, l-phosphinothricyl-l-alanyl-l-alanine (**12**). PPT has been widely used in the agricultural industry, and it is the active ingredient in a number of common herbicides. The compound has been found to inhibit GS from a variety of species, including *Mt*GS, and a selection of these data is presented in Figure 4.

**Figure 5:**
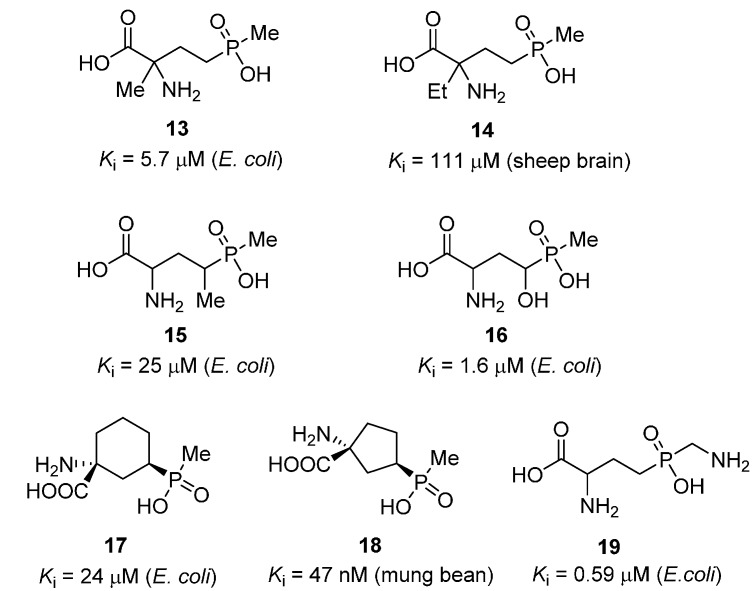
Structures and biological activities of phosphinothricin analogues **13** [26], **14** [37], **15** [26], **16** [43], **17** [26], **18** [38] and **19** [39].

A number of structural analogues of PPT have been prepared and evaluated for their ability to inhibit GS (Figure 5). The main strategies for modification of the PPT scaffold include the introduction of an additional α- or γ-substituent [26,37], cyclization of the carbon backbone [26,38] or modification of the methyl group [39,40,41,42]. The introduction of an α- or γ-alkyl substituent (**13**, **14** and **15**) led to a reduction in potency as compared to the parent compound. In contrast, a γ-hydroxyl group was well tolerated; γ-hydroxyphosphinothricin (**16**) was found to be essentially equipotent with PPT [43]. The investigation of cyclic PPT analogues (**17** and **18**) led to the discovery of a number of novel GS inhibitors; however, no improvement in inhibitory activity compared to PPT was obtained. Replacement of the methyl substituent with a hydrogen or an aromatic residue resulted in a dramatic loss of potency [40]. Computer-aided studies suggested that the methyl group of PPT binds in the vicinity of the ammonium-binding site [39], in contrast to the original structure of the *S. typhimurium* GS [44], but in agreement with the higher resolution structure of *Mt*GS reported eariler the same year [13]. Based on this assumption, Berlicki *et al.* prepared a number of PPT analogues bearing a polar substituent on the methyl group. The most promising compound in this series (**19**) contained a primary amine substituent on the methyl group, and was almost equipotent to PPT.

**Figure 6:**
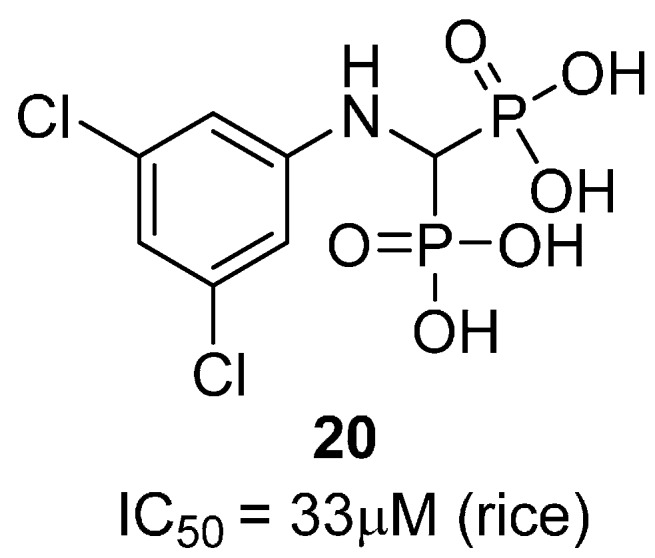
Structure of the most active aminomethylenebisphosphonic acid derivative**.**

Aminomethylenebisphosphonic acid derivatives have been reported as potent GS inhibitors [45]. In this study, 17 compounds were evaluated for their inhibitory activity against plant synthetases, and ten active compounds were identified. Amongst these compound **20**, based on a 3,5-dichlorophenyl scaffold, was found to be the most active (Figure 6). It has been proposed that these compounds do not in fact bind to the glutamate-binding site, but rather interact with the enzyme near the ATP-binding site [45,46], however this claim has not yet been substantiated by structural or assay data.

**Figure 7:**
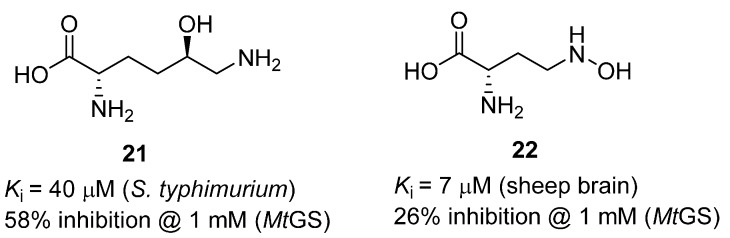
Glutamate analogues found to weakly inhibit *Mt*GS [47].

In 2008, a comprehensive study was undertaken to examine amino acid analogues of MSO and PPT, and mimics of the phosphorylated intermediate, against *Mt*GS [47]. This investigation included a number of compounds known to inhibit GS enzymes from other species, as well as compounds identified from a virtual screening campaign. A number of weak *Mt*GS inhibitors (**21** and **22**) were identified, but none of the compounds was superior to MSO and PPT (Figure 7).

### 2.2. Heterocycles

#### 2.2.1. Purine Analogues as Novel ATP-Competitive Inhibitors

**Figure 8:**
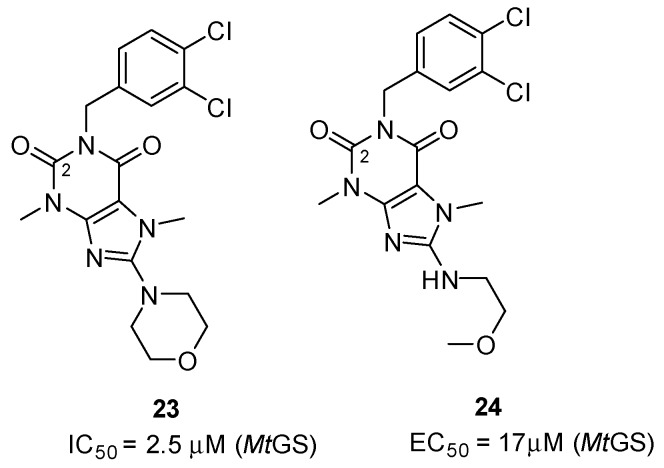
Structure and *Mt*GS inhibitory activity of the diketopurine compounds **23** and **24**.

**Figure 9:**
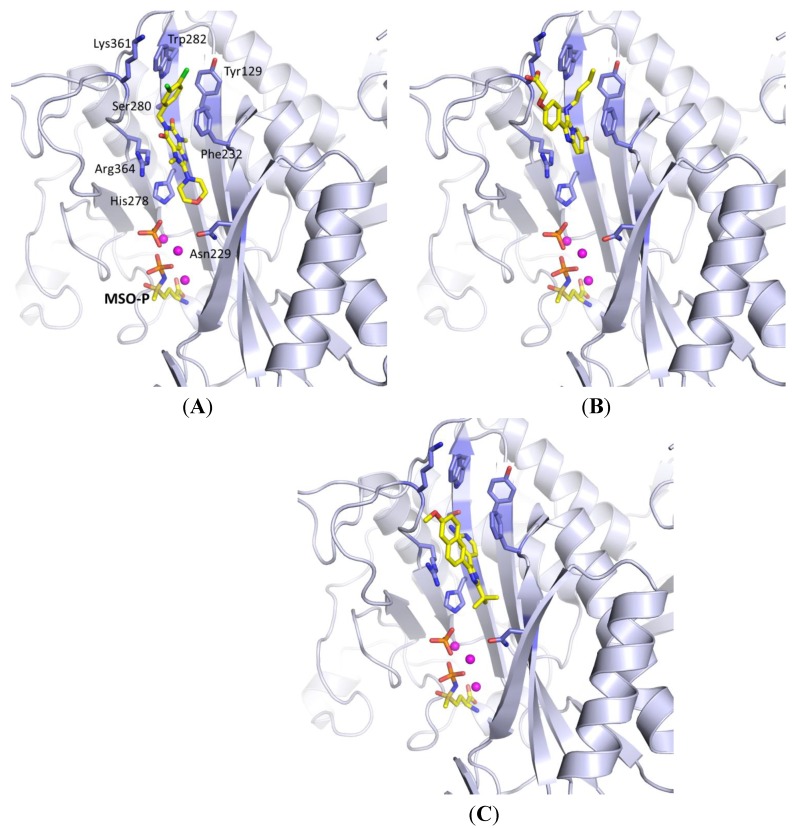
(**A**) X-ray structure of *Mt*GS (blue carbons) bound to purine analogue **23** (yellow carbons), MSO-P, phosphate and magnesium ions (PDB entry 2WHI [20]). (**B**) X-ray structure of compound **32** (yellow carbons) in complex with *Mt*GS (blue carbons), MSO-P, phosphate and magnesium ions (PDB entry 4ACF [49]). (**C**) X-ray structure of **37** (yellow carbons) in complex with *Mt*GS (blue carbons), MSO-P, phosphate and magnesium(PDB entry 3ZXV [50]).

Purine derivatives are among the most widely studied classes of nitrogen-containing heterocycles, and have been utilized in the design of numerous therapeutic agents [48]. As part of a high-throughput screen of AstraZeneca’s corporate library, compounds containing a diketopurine core were identified as ATP-competitive inhibitors of *Mt*GS (Figure 8, **23** and **24**) [20]. The most potent compound of this class (**23**) was shown to inhibit the enzyme with an IC_50_ of 2.5 ± 0.4 µM. In addition, two X-ray structures of **23** in complex with *Mt*GS revealed that this class of inhibitors do indeed bind to the enzyme’s nucleotide-binding site (Figure 9A), and that the mode of binding is essentially the same for both conformers of the enzyme. The interactions were found to be largely non-polar, with only two hydrogen bonds linking the inhibitor and protein. The inhibitor’s morpholino group stacked near the side chain of His278, and a hydrogen bond linked the ring oxygen to the side-chain nitrogen of Asn229. The 8-aminopurine-2,6-dione moiety was stacked between the side chains of Phe232 and Arg364, and the side-chain hydroxyl group of Ser280 donated a hydrogen bond to the oxygen at the C2-carbonyl in the heterocylic core. The dichlorophenyl group was positioned farther out towards the solvent, and interacted with the π-systems in the side chains of Tyr129 and Trp282 (Figure 9A). Compound **23** displayed a 60-fold weaker activity against human GS, validating the nucleotide-binding site as a promising target for the production of selective inhibitors.

#### 2.2.2. 3-Aminoimidazo[1,2-*a*]pyridines

A second class of compounds, the 3-aminoimidazo[1,2-*a*] pyridines, were also identified as novel *Mt*GS inhibitors during AstraZeneca’s HTS campaign. To explore their structure-activity relationships (SAR), two different approaches were employed. In the first study, the pyridine and phenyl ring substituents were varied [28], while in the second, a design matrix was constructed to investigate the amine and phenyl ring substituents [49].

In the first study, 19 compounds containing various pyridine substituents were synthesized and evaluated for inhibitory activity [28]. Iodide and bromide substituents in the 6-position of pyridine ring gave the most potent inhibitors, and compound **25** was selected for further investigation. When the *meta*-hydroxy and *para*-methoxy substituents in compound **25** were interchanged, the resulting compound was found to be inactive, indicating the importance of having a hydrogen-bond donor in the *meta-*position. By removing the *para*-methoxy substituent, an improvement in activity (**26**; IC_50_ = 3.3 μM) was obtained, suggesting that this substituent is not involved in productive interactions with the protein. The importance of the hydrogen bond-donating capacity was further examined by moving the hydroxyl group to the *ortho-* and *para-*positions. Compounds with a hydrogen bond-donating group in the *meta-*position were found to be active, and a considerable improvement was obtained by the introduction of a carboxylic acid at this position. The resulting nanomolar inhibitor (**27**; IC_50_ = 0.38 μM) represents the first reported sub-micromolar inhibitor of *Mt*GS.

**Figure 10:**
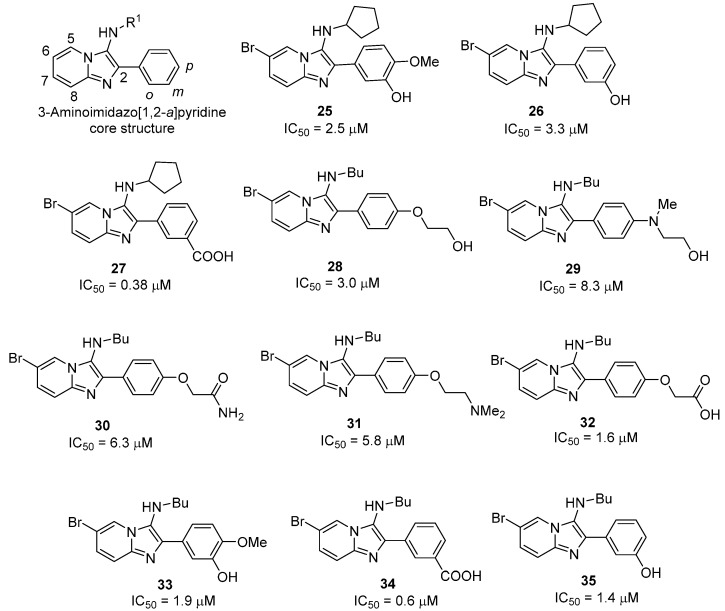
Structure and *Mt*GS inhibitory activity of 3-aminomidazo[1,2-*a*]pyridine analogues.

To further explore the imidazopyridine class of inhibitors, an experimental design was conducted to investigate how size and polarity in four different positions (*ortho*, *meta* and *para* on the phenyl ring, together with R^1^; see Figure 10) influenced activity [49]. Each position was varied independently, by altering the isocyanide and aldehyde building blocks.

A small library consisting of 16 compounds was synthesized and tested for their *Mt*GS inhibitory activity. Of these, compound **28** was found to inhibit the enzyme with an IC_50_ of 4.9 µM. Interestingly, **28** had a different 3-amino substituent (R^1^ = butyl) and lacked the *meta*-hydrogen bond donor previously identified as a requirement for activity. Based on this finding, an investigation of the R^1^-position was undertaken, retaining the 2-hydroxyethoxy substituent in the *para-*position. This revealed a preference for small linear alkyl groups, ideally an *n-*butyl substituent, at this position. The 2-hydroxyethoxy substituent of the lead compound was then replaced by different substituents in the *para*-position or moved to the *meta*-position. Compounds bearing a variety of different polar groups (**29**–**32**) were almost equipotent. To explore the effect of having an *n*-butyl chain instead of the cyclopentyl group in the R^1^ position, compounds **33**–**35** were synthesized. The *n*-butyl and cyclopentyl analogues displayed similar inhibitory activities in all but in one case (**34**, IC_50_ = 0.6 μM, compared to **27**, IC_50_ = 0.38 μM).

In addition, an X-ray structure was obtained with one of the imidazo[1,2-*a*]pyridine based inhibitors (**32**, see Figure 9B) and *Mt*GS in the presence of chemically synthesized MSO-P and magnesium [49]. Interestingly, the electron density was considerable weaker for the 2-phenyl group than for the rest of the inhibitor, indicating that there is some rotational freedom around the bond linking it to the imidazo[1,2-*a*]pyridine scaffold. The NH of the *n*-butylamino group was found to form a hydrogen bond to the backbone carbonyl of Lys361, as well as interact with the side chain OH of Ser280 via a bridging water molecule. The ether oxygen displayed a hydrogen-bond interaction to Asn359, while the carboxylate group interacts with either or both of the side chains of Asn359 or Lys361. An additional hydrogen bond between the side-chain amide oxygen of Asn359 and the main-chain amide nitrogen of Lys361 stabilizes the protein’s conformation and suggests that the side-chain amide nitrogen of Asn359 is most accessible to the ligand.

#### 2.2.3. 2,4,5-Trisubtituted Imidazoles

**Figure 11:**
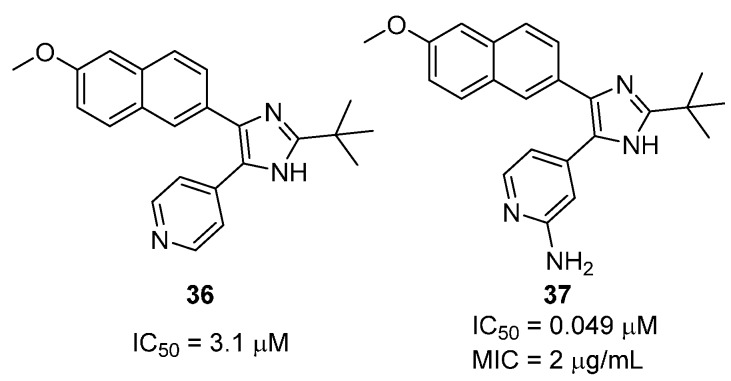
Structure and *Mt*GS inhibitory activity of the 2,4,5-trisubstituted imidazoles **36** and **37**.

Several 2,4,5-trisubtituted imidazoles have also been reported as potent inhibitors of *Mt*GS [50]. In this study, various 2- and 4-substituents were evaluated while keeping the 5-methoxynaphthalene group constant (Figure 11). A *tert*-butyl group was found to be the optimal 2-substituent while a 4-pyridine in the 4-position of the imidazole was essential for inhibition. This was attributed to a hydrogen-bond interaction between the pyridine nitrogen and the side chain of Ser280, an interaction that is equivalent to that of N1 in ATP’s adenine ring. The introduction of a 2-amino group into the 4-pyridyl ring afforded the most potent inhibitor in the series (**37**, Figure 11).

This modification resulted in a 60-fold potency increase compared to the unsubstituted pyridine analogue **36**. An X-ray structure of **37** in complex with *Mt*GS suggested that the enhancement was due to the formation of an additional hydrogen bond between the exocyclic NH_2_ group and the side chain of Ser280 (Figure 9C). Compound **37** is the most potent *Mt*GS inhibitor reported to date, with an IC_50_ of 0.049 μM. More importantly, it was found to be a good inhibitor of bacterial growth, displaying an MIC of 2 μg/mL against *M. tuberculosis*.

#### 2.2.4. Potential ATP-Competitive Inhibitors

**Figure 12:**
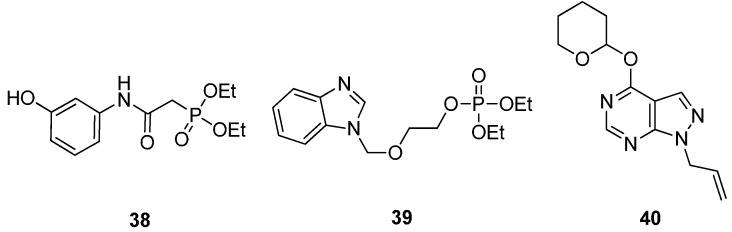
Structure of three classes of potential *Mt*GS inhibitors.

Recently, the synthesis has been reported for a number of proposed *Mt*GS inhibitors (Figure 12), all of which were designed to mimic some structural features of ATP. In total, three classes of such compounds have been described, including phosphonate esters (**38** [51]), phosphate esters (**39** [52]) and allopurinol (**40** [51]) derivatives. However, at the time of writing, no studies describing the biological activity of these compounds have been reported.

Very recently, a patent describing the isolation of two fractions from the twigs of the *Byttneria herbecea* plant exhibiting *Mt*GS inhibitory activity was registered [53]. One of the fractions (fraction K) was found to inhibit *Mt*GS with an IC_50_ value of 4.5 mg/mL and also to inhibit growth *M. bovis* BCG with an IC_50_ value of 1.56 µg/mL. Unfortunately, the structure of the active compound in fraction K was not revealed, and moreover, the large discrepancy between the enzyme and bacterial IC_50_s suggests that it may not attain its antibacterial activity via inhibition of *Mt*GS.

## 3. Conclusions

Though drugs of diverse chemical structures acting through different mechanisms are available for the treatment of *M. tuberculosis*, there are still many problems associated with the currently available compounds. Therefore, medicinal chemists worldwide are designing, synthesizing and evaluating a variety of novel molecules for inhibiting the bacterium’s growth, with a special emphasis on identifying new drug targets. This review presents an overview of the various strategies and compounds utilized to inhibit glutamine synthetase, a promising target for the development of drugs for TB therapy. The currently described inhibitors can be divided into two main classes, those that target the glutamate-binding site, and ATP-competitive inhibitors. Compounds belonging to the first class are typically low MW and highly polar analogues of glutamate, methionine sulfoximine or phosphinothricin. Moreover, the SAR surrounding this inhibitor class has been found to be restricted; phosphinothricin remains the most potent member of this class. In contrast, the ATP-competitive inhibitors are generally larger, more hydrophobic and more drug-like. Furthermore, this type of compound shows greater promise for drugs that selectively target the bacterial enzyme. Significant effort has been directed towards uncovering the SAR of the lead compounds in this class, resulting in the identification of nanomolar potent *Mt*GS inhibitors with good *in vivo* antibacterial activities. Future studies should focus on the development of more potent compounds with sub-micromolar MIC values. We foresee this as a promising strategy for the development of new anti-TB drugs.
